# Pore Geometry Optimization of Titanium (Ti6Al4V) Alloy, for Its Application in the Fabrication of Customized Hip Implants

**DOI:** 10.1155/2014/313975

**Published:** 2014-10-21

**Authors:** Sandipan Roy, Debojyoti Panda, Niloy Khutia, Amit Roy Chowdhury

**Affiliations:** ^1^Department of Aerospace Engineering and Applied Mechanics, Indian Institute of Engineering Science and Technology, Shibpur 711103, India; ^2^Department of Civil Engineering, Indian Institute of Engineering Science & Technology, Shibpur, West Bengal, India

## Abstract

The present study investigates the mechanical response of representative volume elements of porous Ti-6Al-4V alloy, to arrive at a desired range of pore geometries that would optimize the reduction in stiffness necessary for biocompatibility with the stress concentration arising around the pore periphery, under physiological loading conditions with respect to orthopedic hip implants. A comparative study of the two is performed with the aid of a newly defined optimizing parameter called pore efficiency that takes into consideration both the stiffness quantity and the stress localization around pores. To perform a detailed analysis of the response of the porous structure over the entire spectrum of loading conditions that a hip implant is subjected to* in vivo*, the mechanical responses of 3D finite element models of cubic and rectangular parallelepiped geometries, with porosities varying over a range of 10% to 60%, are simulated under representative compressive, flexural as well as combined loading conditions. The results that are obtained are used to suggest a range of pore diameters that lower the effective stiffness and modulus of the implant to around 60% of the stiffness and modulus of dense solid implants while keeping the stress levels within permissible limits.

## 1. Research Background

This study is part of a collective research effort of the Orthopedic Biomechanics Research Group of the Department of Applied Mechanics and Aerospace Engineering that has been focusing on the overall designing of patient-specific, modular, hip implants using porous Ti-6Al-4V alloy. Earlier studies include designing a fully solid customized hip implant (with no porosity) and studying the zonal stress and strain responses under actual physiological loading conditions [1], both experimentally and computationally, to identify the regions in the implant where porosity is going to be introduced subsequently.

## 2. Introduction

In recent times, titanium and its alloys have been put into broad usage as the preferred biomaterial for orthopedic implants owing to its desirable mechanical and biological properties [2–5]. However, despite its proven medical success, technical shortcomings in the form of implant loosening and stress shielding have arisen, leading to the reduced longevity of the implant by its aseptic loosening and necessitating frequent revision surgeries in the future. This has been primarily attributed to the substantial mismatch that arises between the stiffness of the bone and that of the titanium [6] alloy that constitutes the implant which gives rise to a reduction of stresses transferred to adjoining bone leading to bone resorption. In view of this problem, porous titanium has been suggested as an improved alternative because of its advantages in the form of lower density and hence effective stiffness along with high impact energy absorption capacity owing to its porous structure [7, 8]. In addition to this, the material also allows for bone ingrowth into the porous structure leading to an enhanced stability at the bone-implant interface [6].

By increasing the porosity in the implant material, appreciable reduction in the relative density and consequently effective stiffness is obtained [9]. But the process of attenuating the aforementioned mismatch in stiffness by way of increasing the percentage of porosity has revealed that for higher porosities, intense stress localization occurs in the implant around the pore periphery, which leads to the reduction of the mechanical durability of the material, despite its enhanced compatibility with the surrounding bone material [10]. This counteractive action could become detrimental to the structural stability of the material by potentially reducing the damage tolerance of the implant, especially under accidental loading conditions, thereby inducing local failure at the high stress localized regions leading to premature failure. With the octahedral model, the mechanical behavior is investigated for the component of these materials under shearing loads, the loading state of struts within the porous body is analyzed for these materials under torsion, and the mechanical performance is further discussed for these materials under bending moment based on their properties of uniaxial tension and compression [11–13].

In order to improve the functionality and structural compatibility of the titanium implant besides addressing the issue of stress concentration, optimization of the stiffness and stress concentration needs to be done in a systematic and detailed manner. In view of this requirement, the present study aims to analyze, through finite element analysis, the linear elastic mechanical response of representative cubic and rectangular parallelepiped volume element models of porous Ti-6Al-4V alloy upon being subjected to representative compressive, flexural, and combined loading action that is typically experienced by orthopedic hip implants* in vivo*. To capture the mechanical response of the porous alloy over a wide range of porosity, the porosity percentages of these models are varied from low porosity, 10% and 20%, to medium porosity, 30% and 35%, to high porosity, 60%. Now, since it is very difficult and time-consuming to model and analyze the whole hip implant for all the different range of porosities and pore diameters that are tested at the desired level of accuracy, owing to the very high number of nodes and elements that gets generated in the process, representative volume elements (containing lesser number of nodes of cubic (for compressive loading response) and rectangular parallelepiped geometries (for flexural and combined response) are used as an alternative. The diameters of the spherical pores that are finally suggested in this paper are going to be applied during the fabrication of porous customized hip implants for Indian and subcontinental anatomical structures by the laser-engineered net shaping (LENS) technique. Disconnected (closed) pores with square array distribution pattern that are spherical in shape are maintained throughout the volume for the cubic and rectangular parallelepiped geometries, owing to the relative ease and precision of the fabrication of spherical pores using the LENS technology. Details of the computational modeling procedure have been detailed in the subsequent sections on modeling and methodology.

## 3. Modeling and Methodology

In order to obtain a realistic simulation of the performance of the porous structure under representative physiological loading conditions, the procedure of finite element modeling and analysis is adopted instead of previously investigated analytical methods [14–16]. This is because the analytical methods are found to employ simplified assumptions and are unable to furnish a full-field solution of the response of the structure under physiological loading conditions.

### 3.1. 3D Modeling

**Figure 1:**
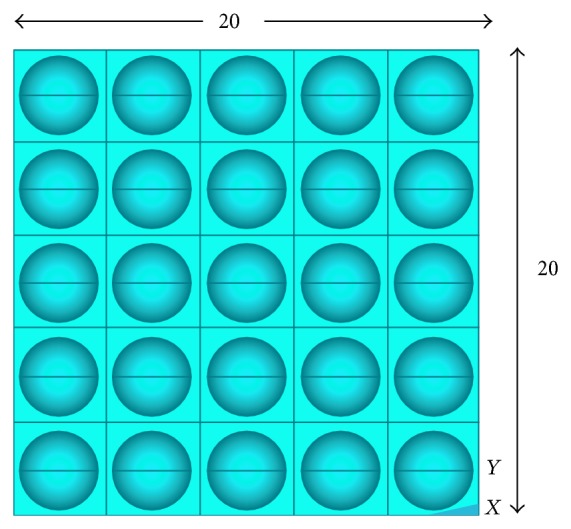
Pore distribution pattern (square array).

In order to investigate the response of the porous structure under compressive loading, twenty-five cubes, each of side 20 mm, are modeled computationally. Porosities ranging from 10% to 60% as aforementioned are introduced into the cubes in the form of uniformly spaced disconnected spherical holes conforming to the square array pore distribution pattern as illustrated in Figure 1. Keeping the porosity constant, five models with increasing pore diameters are modeled for each of the five porosity percentages considered, as has been shown in Table 1.

**Figure 2:**
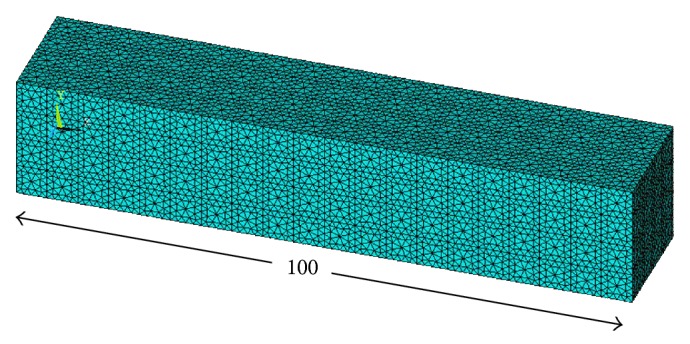
A typical meshed rectangular parallelepiped beam model.

In order to investigate the flexural and combined loading response of a representative volume element of the porous titanium implant for all the different combinations of porosity and pore diameters, twenty rectangular parallelepiped finite element models, with pore diameters as listed in Table 1, are modeled. Each of these rectangular parallelepiped models has a length of 100 mm and a square cross-section of edge length 20 mm (Figure 2). Porosities ranging from 10% to 60%, as aforementioned, are introduced into the models in the form of uniformly spaced spherical pores conforming to the square array pore distribution pattern, as has been illustrated in Figure 1. For a given porosity, four different pore diameters are considered (excluding the smallest pore diameter listed in Table 1 for each porosity). The pore diameters are determined in a manner that ensures uniform pore distribution with no pore coalescence or connectivity in the representative volume elements. The diameters of the spherical pores are restricted to a minimum of 1.65 mm to address the shortcoming that, with decreasing diameter, the possibility of imperfections and resulting deviation of the pore morphology from the spherical shape increases, when they are fabricated by the LENS technology. The material properties assigned to all the finite element models conform to that of the Ti-6Al-4V alloy (Young's modulus, *E*_0_: 117 GPa, and Poisson's ratio: 0.33). The behavior of the material is configured to be linear, elastic and isotropic in nature for the purpose of finite element computation.

Two fully dense solid models, having geometries identical to that of the cubic and rectangular parallelepiped porous models, are also prepared and analyzed under loading and boundary identical to that of the corresponding porous models to serve as the control for the computation of the relative stress and stiffness parameters that are going to be used for the purpose of optimization and determination of relative reduction in stiffness that is achieved through the incorporation of porosity. Furthermore, they help in making the relative stress and stiffness parameters independent of the magnitude of loading to which the system is subjected to, thereby improving the usability of the results for all kinds of bone implants.

### 3.2. Meshing

While meshing the 3D models for finite element computation using the ANSYS 14 Mechanical APDL software, the element type is chosen to be SOLID187, owing to the suitability of this 3D, 10-node, tetrahedral structural solid element for modeling irregular meshes (such as those produced from various CAD/CAM systems) and for possessing quadratic displacement behavior. An optimum edge length of 0.5 mm is chosen for elements around the pore boundaries as the stress localization at these sites needs to be accurately quantified for proper estimation of stress concentration parameters. A larger element size of 1 mm is chosen for the elements at all the other locations bearing in mind the substantial time taken for one complete finite element analysis and solution of a porous model.

## 4. Loading and Boundary Conditions

### 4.1. Boundary Conditions

**Figure 3:**
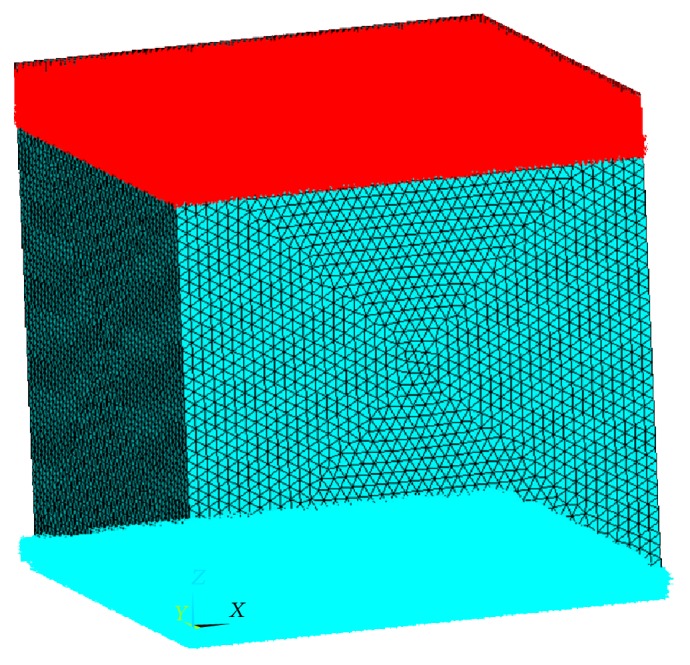
Applied compressive loading distributed over the top surface (shown in red) and bottom surface fixed for all degrees of freedom (shown in blue) on a typical porous cubic model subjected to compressive loading only.

In the case of investigating the pure compression response of the porous structure, the lower surface of the porous cubic models is fixed in all degrees of freedom. The complete fixity at the bottom surface ensures that the nodes at this region undergo zero displacement under the compressive loading action (Figure 3).

**Figure 4:**
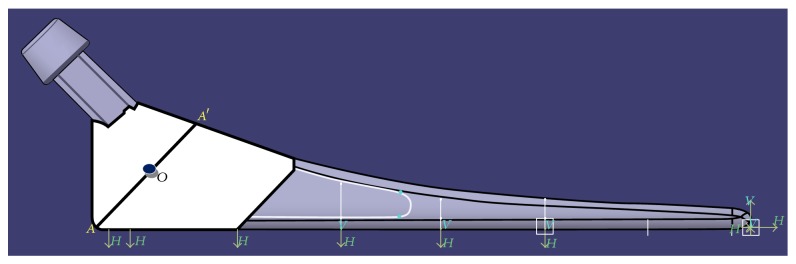
The designed customized hip implant [1] that is being used as the standard for our current investigation, with the demarcation of the region where porosity is going to be introduced (region of interest) shown in white.

**Figure 5:**
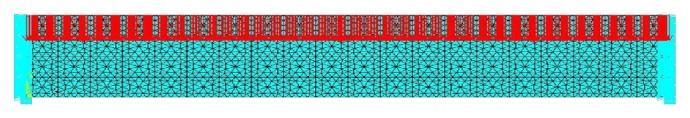
Applied uniformly distributed load on the top longitudinal surface and fixed boundary condition on the two ends of a typical porous beam model.

While applying the boundary conditions for the case of flexural response, it has been assumed that a rectangular parallelepiped porous model of a given porosity and pore diameter, henceforth to be referred as a porous beam model, is a representative volume element (RVE) of the portion of the hip implant where that given porosity is going to be introduced in the form of spherical pores in square array distribution pattern. The region of the hip implant where porosity is going to be introduced, making it the region of interest for the current investigation, has been demarcated in Figure 4. In order to simulate the transmission of all kinds of forces and moments between the elements in the portion below the neck of an implant under real life loading, the two ends of the porous beam models are idealized to be fixed in all degrees of freedom for each of the models that are tested for flexure (Figure 5).

**Figure 6:**
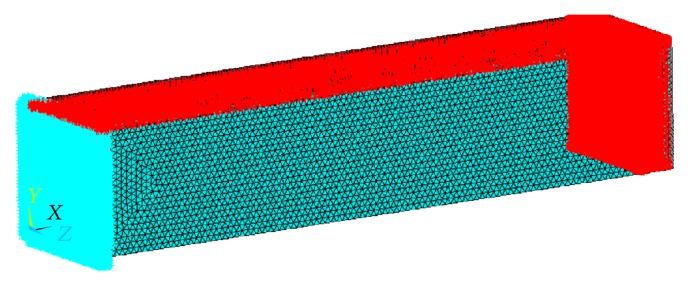
Applied uniformly distributed load on the top longitudinal surface and compressive loading distributed over one end (shown in red) of a typical porous cantilever beam model subjected to combined loading. The other end of the model is fixed for all degrees of freedom (shown in blue).

Lastly, in order to investigate the combined response of the porous beam models under the simultaneous action of representative transverse and compressive loading, only one end of each of the porous beam models is assigned to be fixed in all degrees of freedom (Figure 6). This allows the compressive load to be applied at the other end in the longitudinal direction while the transverse load gets applied on the top surface of the beam to eventually give rise to a combined axial compressive and cantilever loading action.

### 4.2. Loading

Since our current series of investigations addresses the design criteria of the customized hip implant that has been designed as per [1], a close simulation of the stresses that arise due to the bending action owing to the eccentricity of the physiological load acting on the head of the implant has been attempted for. The muscle force components which were used for this purpose were obtained from the hip joint contact forces that are transmitted in case of 30% gait cycle as is available in the literature [18]. For this purpose, a portion of the designed solid implant finite element model below the neck region of the implant was substructured after the application of muscle force components on the head of the implant. It was found from the investigation detailed in [1] that a maximum von Mises stress of magnitude 93 N/mm^2^ arises near the region below the neck of the implant where porosity is supposedly going to be introduced, under the application of real life loading on the head of the implant.

With the aim of producing the same value of compressive stress on the intermittent fully dense solid cross-sections of the porous cubic models that are being tested for compression only, a compressive load of magnitude 37200 N (= 93 × 20 × 20) is distributed uniformly over the nodes on the top surface of the cubes with their bottom end fixed in all degrees of freedom.

**Figure 7:**
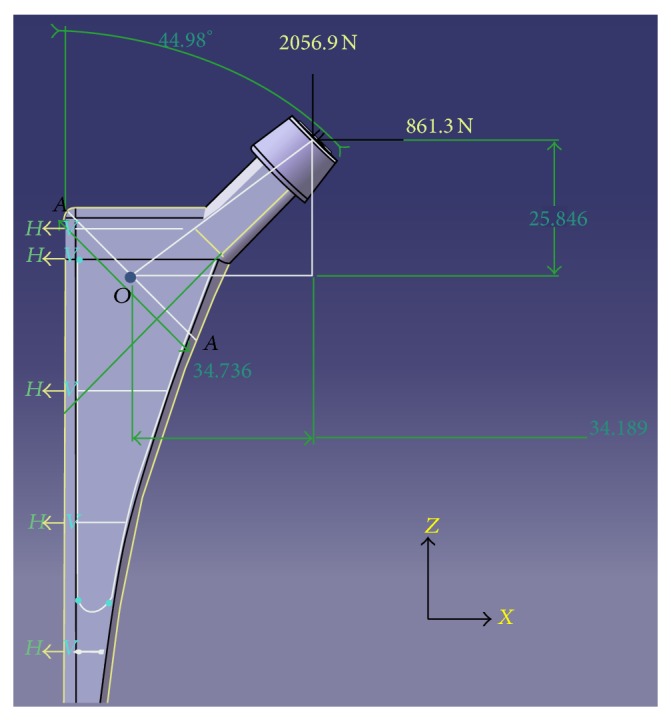
Muscle force components as per [17] acting at the head of the designed fully solid, customized, hip implant [1].

For the computation of the maximum moment that is to be applied on the porous beam models, the resultant moment acting in the *XZ* plane at the midpoint, *O*, of the section, *AA*′, of the implant (Figure 7), due to the force components in the *X* and *Z* directions, has been calculated. The section *AA*′ has been chosen for this purpose because it lies approximately at the middle of the region where porosity is supposedly going to be introduced, as is shown in Figure 4. Similarly, since the exact location of the point through which the neutral axis is passing is not known, it has been assumed that the same passes through the midpoint of the section *XZ*, through point *O*. A careful examination of the loading as described in Figure 7 then reveals that a resultant moment of magnitude 48100 N-mm acts at the point *O* of cross-section *AA*′.

Although the bone into which the implant is going to be inserted, that is, femur, is itself oriented at an angle of approximately 14.5° with the vertical* in vivo*, the same has been assumed to be oriented vertically for the purpose of moment computation above. As the resultant moment gets effectively reduced in magnitude due to the inclination, our assumption is on the conservative side.

The bending stress that arises due to the moment of 48100 N-mm, at the extremity *A*′ of the cross-section, is subsequently computed analytically to be around 18.1 N/mm^2^. This value of the maximum bending stress is then equated with the maximum bending stress that must arise at the mid-span of a rectangular parallelepiped solid beam model (with no porosity and both ends fixed) to arrive at the required loading intensity of 60.5 N/mm. A uniformly distributed load of intensity 60.5 N/mm is then applied on the top longitudinal surface of the beam acting in the transverse direction as shown in Figure 5. However, it is to be remembered here that since only relative terms are going to be used for the purpose of optimizing stress and stiffness quantities in determining the design pore diameter, the absolute values of the applied loading on the porous beam models do not as such carry any significance other than simulating the bending response of the porous structure in a realistic manner.

Since the realistic loading of the implant is a combination of the compressive and flexural loading cases, the same is finally applied on the porous beam models after the flexural analysis by replacing the fixed boundary condition on one of the two end cross-sections of the beam with distributed load producing longitudinal compression. A transverse loading in the form of uniformly distributed load is also applied on the longitudinal top surface of the beam to produce simultaneous cantilever action.

In superimposing the two types of loading, the magnitude of the compressive load is kept the same at 37200 N to produce a compressive stress of 93 N/mm^2^ at a fully solid cross-section of the beam due to axial compression. The extreme fiber bending stress of 18.1 N/mm^2^ that is obtained analytically due to the moment acting at point *O* of the region of interest is then equated with the maximum bending stress that must arise at an intermittent fully solid cross-section at the middle of the cantilever porous beam model to arrive at the required loading intensity of 20.16 N/mm that is distributed as uniformly distributed load over the longitudinal top surface of the beam, as shown in Figure 6.

A set of analyses, with loading and boundary conditions identical to that applied to the porous beam models, is performed with the fully dense solid rectangular parallelepiped model since it is to be used as the control for calculating the relative stiffness quantities and stress concentration parameters.

## 5. Postprocessing

### 5.1. Compressive Loading

After simulation, the deflection of the central node on the top surface of each of these cubes on which the external compressive load acts, is noted down as an indirect measure of the effective modulus, *E*_eff_, of the porous models, *E*_eff_ being inversely related to the noted deflection. The relative modulus, *E*_*rel*⁡_, is then calculated by the relation, *E*_*rel*⁡_ = *E*_eff_/*E*. Since our investigation necessitates the determination of the stress concentration at the sites of high stress localization in the models, the same is done by recording the maximum von Mises stress, *σ*_*c*,porous_, that is generated adjacent to the peripheries of the pores for each of these models. This is done by observing the stress contour obtained at a section cut at right angles to the direction of loading, which is located midway between the planes of applied external loading on top and fixed boundary at the bottom and intersects through the middle of the pores (as is shown in Figure 5).

The relative stress concentration between the porous models and the fully dense solid model is then quantified in terms of the stress concentration factor for compressive loading, *σ*_*c*_. For each of the porosities and pore diameters, *σ*_*c*_ is calculated by the relation *σ*_*c*_ = *σ*_*c*,porous_/*σ*_*c*,solid_, where *σ*_*c*,solid_ is the von Mises stress in a section located parallel to the loading plane at the middle of the solid dense model.

### 5.2. Flexural Loading

After completion of the finite element analysis for flexural loading, a critical transverse section intersecting through the middle of the pores at around the mid-span of the porous beam model is cut and the highest von Mises stress, *σ*_*b*,porous_, which is generated around the pore periphery at that section, is recorded (Figure 7). The stress concentration factor for flexural loading, *σ*_*b*_, for each of the porosities and pore diameters is then calculated by the relation *σ*_*b*_ = *σ*_*b*,porous_/*σ*_*b*,solid_, where *σ*_*b*,solid_ is the corresponding observed maximum von Mises stress at the mid-span for the fully dense solid beam model. The deflection of the central node on the top surface of the beam is then observed from the numerical results for deformation along *y*-axis to obtain an indirect measure of the bending stiffness of the porous beam model, as is detailed in Section 6.

### 5.3. Combined Loading

After the finite element simulation of the porous cantilever models is completed for the porous beam models, the von Mises stress generated at the middle span of the cantilever around the pore peripheries near the bottommost fibre, *σ*_porous_, is recorded. As the bottommost pore peripheries experience the maximum compressive stress due to the superposition of the flexural and axial compressive loading, the same is chosen as the site for determining the stress concentration factor, *σ*_*o*_ = *σ*_porous_/*σ*_solid_ where *σ*_solid_ is the maximum compressive stress generated at the bottommost fibre of the corresponding cross-section for the fully dense solid cantilever model under combined loading. The deflection of the central node on the free end surface of the cantilever is then recorded in the longitudinal and transverse direction to obtain an indirect measure of the stiffness parameters of the cantilever model, as is detailed in the following section dedicated to results and discussion of the analysis.

## 6. Results and Discussion

### 6.1. Compressive Loading Response

**Figure 8:**
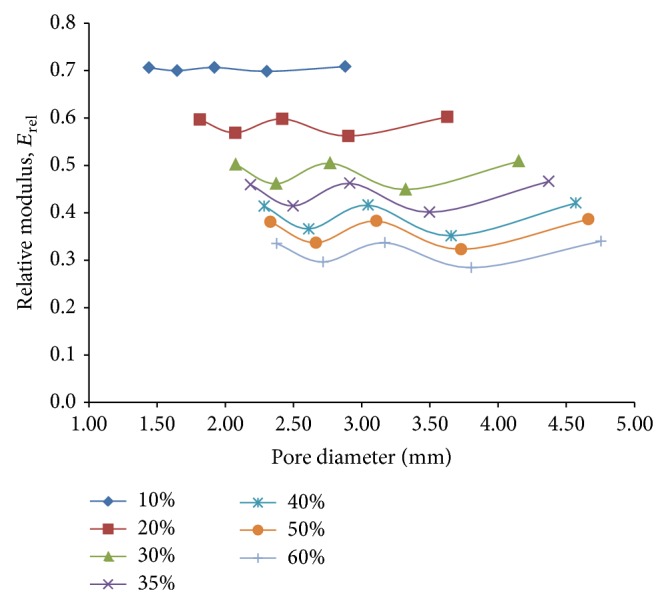
Plots depicting variation of relative modulus, *E*_*rel*⁡_, with pore diameter for different percentages of porosity, using 3D porous FE cube models under compressive loading only.

On analyzing the plots of relative modulus, *E*_*rel*⁡_ against pore diameter for the considered porosity percentages as presented in Figure 8, it was found that, for lower porosities, relative modulus is independent of the pore diameter. But with increasing porosity, this independent variation shifts to more of a nonlinear zig-zag variation. However, even for higher porosities (around 60%) this variation is well within 15% of the mean value of relative modulus. As expected, the relative modulus decreases as the porosity increases and for a porosity of 60%, relative modulus of around 35% is achieved for pore diameters in the proximity of 3.6 mm. This is found to be in congruence with the findings of the previous investigations by Niu et al. [18] where the relative macromodulus was studied against the relative densities of titanium foam samples.

**Figure 9:**
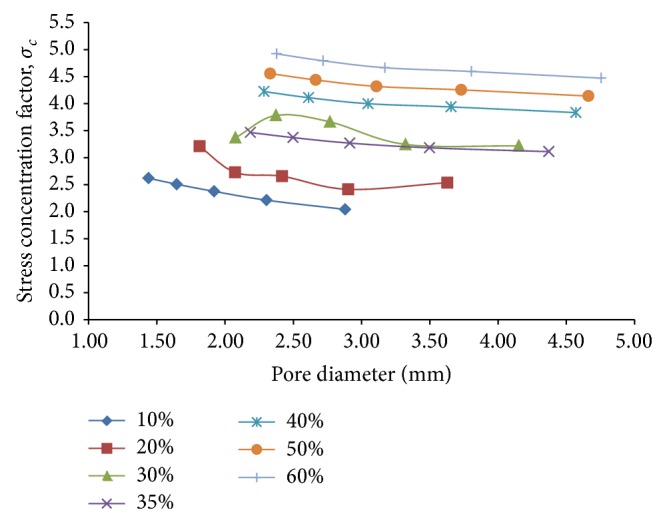
Plots depicting variation of the stress concentration factor, *σ*_*c*_, as a function of pore diameter for different percentage of porosity for the case of compressive loading only using 3D porous FE cubic models.

The relative stress concentration between the porous models and the fully dense solid model, as measured by the stress concentration factor, *σ*_*c*_, is found to decrease consistently with increase in pore diameter for a given porosity (Figure 9). For lower porosities (10% porosity), this reduction is found to be proportionately more at around 21% compared to the higher ones (35% and 60% porosity), for which it is around 9% to 10%. The maximum stress concentration factor for a given porosity is also found to increase appreciably from 2.61 to 4.22, with increase in porosity from 10% to 60%.

**Figure 10:**
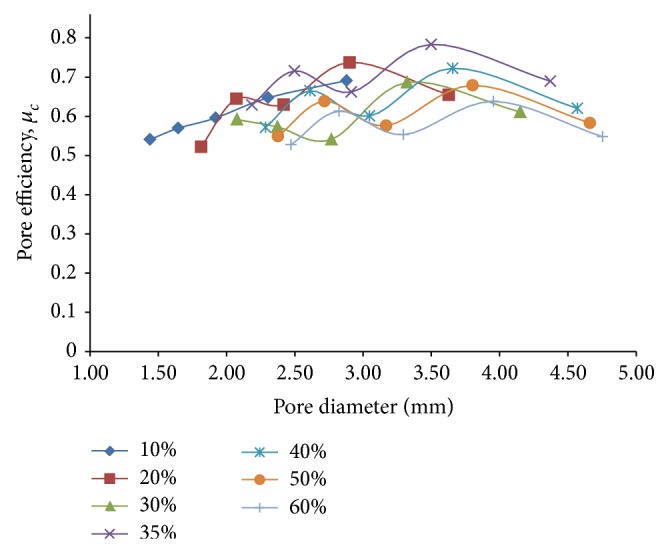
Plots depicting variation of pore efficiency under compressive loading, *μ*_*c*_, with pore diameter, for different percentages of porosity for 3D porous FE cubic models subjected to compressive loading only.

In order to optimize the effects of reduction in effective modulus and stress concentration as aforesaid, a new parameter called pore efficiency for compressive loading, *μ*_*c*_ = 1/*σ*_*c*_ *E*_eff_, is introduced. As it is observed in Figure 10, the pore efficiency (*μ*_*c*_) exhibits an overall increase in value, with some fluctuations in between, against an of pore diameter for a given porosity. This result evidently implies that for a particular porosity percentage, a higher diameter of pores (i.e., a lesser number of holes per unit volume) ensures a greater favorability in terms of stress condition and at the same time, allows us to attain a substantial compatibility with the elastic modulus of the bone. In our entire investigation pertaining to compressive loads, maximum pore efficiency is obtained for a pore diameter of around 3.5 mm corresponding to 35% porosity. This diameter may thus be suggested as an optimum pore diameter for usage in a porous titanium implant with the same spherical pore distribution, from the compression point of view, as it allows for considerable reduction in the effective modulus (around 60%), while keeping the compressive stress concentration factor (at around 3.2) within lower levels. This claim can be further substantiated by the fact that for the case of the designed fully dense solid Ti-6Al-4V hip implant subjected to physiological loading [1], a maximum von Mises stress of magnitude 93 MPa approximately is generated at regions around the neck of the implant when it is subjected to real life loading. Considering that a similar stress concentration would occur in the neck region of the porous implant having a similar shape as the designed fully dense solid implant [1] with pore diameter as suggested above, the maximum von Mises stress that is expected to get generated can be estimated to be around 298 MPa. On taking the compressive yield strength value of annealed Ti-6Al-4V at room temperature to be around 895 MPa, an approximate factor of safety of 3 is thus obtained for the suggested pore diameter. This is found to be fairly on the conservative side, considering the fact that the maximum compressive stresses that were generated during the finite element investigation by Chatterjee et al. [1] were under the loading conditions of normal gait [17]. If a person stumbles while walking, peak muscle forces approximately twice as high as the hip joint contact forces as mentioned in [19] may arise which might induce premature failure for factors of safety lesser than 2.

### 6.2. Flexural Loading Response

**Figure 11:**
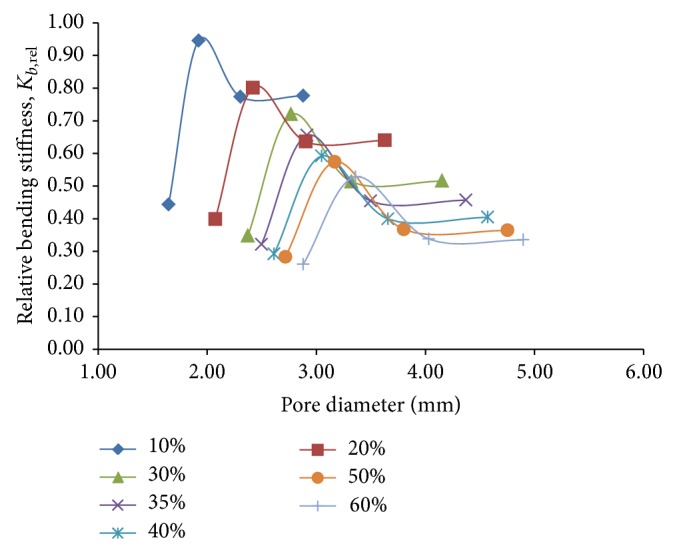
Plots depicting variation of relative bending stiffness, *K*_*b*,*rel*⁡_, against pore diameter for different percentages of porosity for 3D porous FE beam models subjected to flexural loading only.

While investigating the flexural behavior of porous titanium using the beam models, bending stiffness *K*_*b*_ is used as the stiffness quantity for comparing the mechanical performance of the beam models since it mathematically incorporates the section modulus of the cross-section, which is one of the prime governing factors while designing for flexure. Bending stiffness, being inversely related to the deflection of the beam, is indirectly measured from the relation, *K*_*b*_ = Force/Deflection, for both the porous and the fully solid beam models. Since we are only interested in the degree of reduction in stiffness that is achieved with the porous model in comparison to the solid model, relative bending stiffness, *K*_*b*,*rel*⁡_ = *K*_*b*,porous_/*K*_*b*,solid_ (*K*_*b*,porous_ and *K*_*b*,solid_ being the bending stiffness of the porous and solid beam model, resp., under flexural loading only), has been used as the yardstick to measure the reduction in stiffness for the models corresponding to each porosity and pore diameter. As is expected, the curves showing the variation of *K*_*b*,*rel*⁡_ (Figure 11) for each of the porosities show a downward shift with increasing porosity, thereby indicating that the stiffness gets reduced as a whole with increasing porosity. For each of the given porosities, it is observed that the variation of the relative bending stiffness first shows a sharp increase, attains a maximum for a particular pore diameter, and then drops with a lesser gradient to finally become independent of the pore diameter after a certain value. It may be concluded from the plots that for pore diameters of 3.6 mm onwards, constancy in the value of relative bending stiffness is attained for porosities up to 60%.

**Figure 12:**
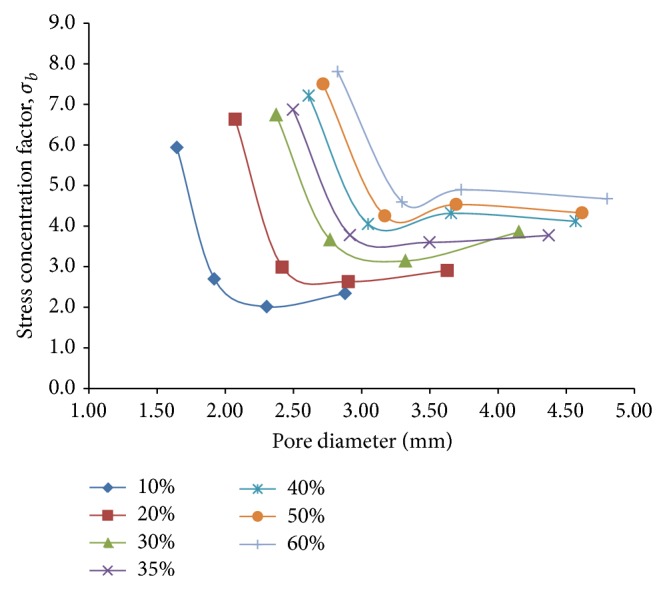
Plots depicting variation of stress concentration factor, *σ*_*b*_, against pore diameter for different percentages of porosity for 3D porous FE beam models subjected to flexural loading only.

The relative stress concentration between the porous models and the fully dense solid model that is quantified in terms of the stress concentration factor for flexural loading, *σ*_*b*_, is found to drop sharply initially with increasing diameter (Figure 12) for a given porosity, attaining a minimum at a certain diameter after which it goes on increasing at a nominal rate. This suggests that for pore diameters beyond 3 mm in the given range of porosities, stress concentration gets substantially reduced and stabilized to be considered for design purposes.

**Figure 13:**
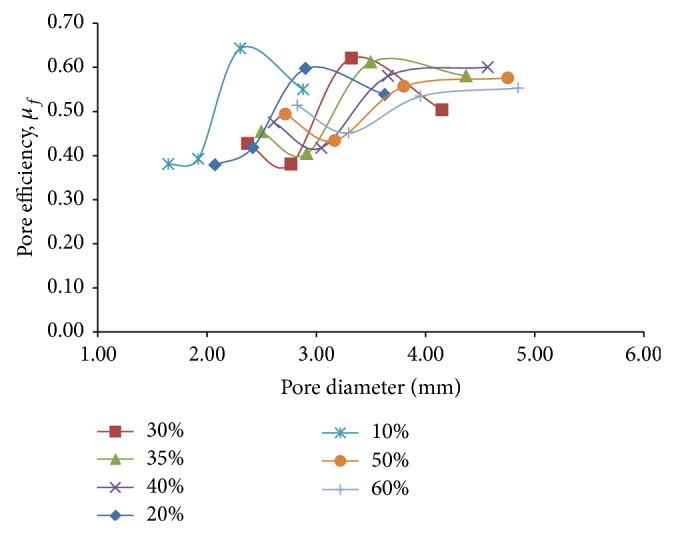
Plots depicting variation of pore efficiency under flexural loading, *μ*_*f*_, with pore diameter, for different percentages of porosity for 3D porous FE beam models subjected to flexural loading only.

In order to optimize the effects of reduction in bending stiffness with the increasing stress concentration factor as aforesaid, a new parameter called pore efficiency for flexural loading, *μ*_*f*_ = 1/(*σ*_*b*_ *K*_*b*,*rel*⁡_), is introduced. Interestingly, it is found that the maximum values of pore efficiency that is attained for each of the different porosity percentages are in close proximity at a value of around 0.6 (Figure 13).This suggests that as we increase the porosity, the maximum values of relative bending stiffness and stress concentration factor decrease and increase, respectively, by equal proportion, as a result of which their net cumulative effect manages to remain more or less unchanged in terms of suitability. Thus, depending on the degree of stiffness reduction that is required to be achieved for perfect compatibility with the bone, a trade-off has to be made between the two conflicting parameters of relative bending stiffness and allowable stress concentration. Since stiffness reduction is the primary design criterion while designing our porous hip implant, a pore diameter in the range of 3.65 mm for 60% porosity is thus suggested as the optimum range of pore diameter. This pore diameter allows for up to 60% reduction in bending stiffness (Figure 11), while limiting the flexural stress concentration factor within 4.35 (Figure 12). Subsequently, from the investigation carried out by Bergmann et al. [19], it can be understood that a maximum von Mises stress of around 605 MPa is expected to be attained around the pore periphery near the neck of a 60% porous hip implant having the same shape as the designed solid hip implant under normal gait conditions for the above stress concentration of 4.35. On taking the yield strength value of annealed Ti-6Al-4V alloy under room temperature conditions to be around 895 MPa, an approximate factor of safety of 2.2 is thus obtained for the suggested value of pore diameter. Since while stumbling action, peak muscle forces as high as 2 times the normal hip joint contact forces might arise approximately [19], this factor of safety is found to be well on the conservative side.

### 6.3. Combined Loading Response

In order to obtain a quantitative measure of the resultant stiffness of the porous cantilever beam model under the combined action of flexural and compressive loading cases, a new mathematical quantity called relative overall stiffness, *K*_*o*_, is introduced. This quantity, *K*_*o*_, which couples the effect of both bending and compression, is defined as the root-mean-square (RMS) value of the relative compressive stiffness under combined loading, *K*_*c*,combined_, and relative bending stiffness under combined loading, *K*_*b*,combined_, and is expressed in the form of

(1) $$\begin{array}{c} K_{o}=\sqrt{\frac{{K_{c,\text{combined}}}^{2}+{K_{b,\text{combined}}}^{2}}{2}}. \end{array}$$

Similar to the computation of the relative bending stiffness under flexural loading only, *K*_*b*,*rel*⁡_, the absolute stiffness of the porous and fully dense solid cantilever beam models in the longitudinal (*x*) and transverse (*z*) direction, is first computed by taking them as the inverse of the corresponding deformation in the *x* and *z* direction, respectively. Since our primary objective lies in the degree of reduction in stiffness that is achieved with the porous titanium models compared to the fully dense solid model, the relative stiffness quantities are found to be of greater relevance for our design purpose. Consequently, the relative compressive (*K*_*c*,combined_) and bending stiffness (*K*_*b*,combined_) under combined loading, in the *x* and *z* direction, respectively, are calculated by the relations

(2) Kc,combined=(Stiffness  of  the  porous  model  in  the  x  direction  under  combined  loading) ×(Stiffness  of  the  solid  model  in  the  x   direction  under  combined  loading)−1,Kb,combined=(Stiffness  of  the  porous  model  in  the  z  direction  under  combined  loading) ×(Stiffness  of  the  solid  model  in  the  z   direction  under  combined  loading)−1.

Here, it must be noted that since, in the computation of the relative stiffness quantities, *K*_*c*,combined_ and *K*_*b*,combined_, the force values, being equal for both the solid and porous models, get cancelled out from the numerator and denominator, the same have been left out beforehand while calculating the absolute stiffness quantities, with no effect whatsoever. As a consequence, it may further be stated that although the values of the forces that are applied for simulation purposes have been obtained from the muscle force components at the hip joint [18] for the design of a hip implant, they bear no significance in determining the relative stiffness quantities. Also, since within the elastic zone, the localized stress levels at the pore periphery are linearly related to the load applied for a given geometry, the stress concentration factor (computed as the ratio of the maximum stress generated in the porous and solid models) is actually independent of the loads that are applied on the representative porous and solid volume element models.

**Figure 14:**
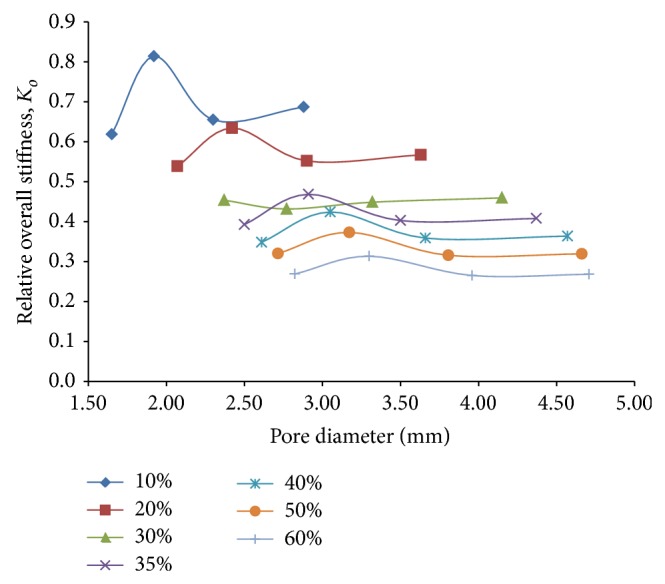
Plots depicting variation of relative overall stiffness, *K*_*o*_, against pore diameter for different percentages of porosity of 3D porous FE cantilever beam models subjected to combined loading action.

Similar to the curves showing the variation of relative bending stiffness under flexural loading, *K*_*b*,*rel*⁡_, in Figure 11, the relative overall stiffness, *K*_*o*_, shows a downward shift with increasing porosity (Figure 14), thereby proving that the stiffness does get reduced as a whole with increasing porosity percentages, as is expected. The plot suggests that for a given porosity, the overall stiffness first shows an increase and attains a maximum against a particular pore diameter. With further increase in pore diameter for a given porosity, the overall stiffness decreases gradually to eventually become independent of the pore diameter after a certain point. As per Figure 14, it may be concluded that for a porosity of 60%, overall stiffness as low as 35% of the corresponding stiffness of the fully dense solid model could be achieved corresponding to a pore diameter in the proximity of 3.65 mm.

**Figure 15:**
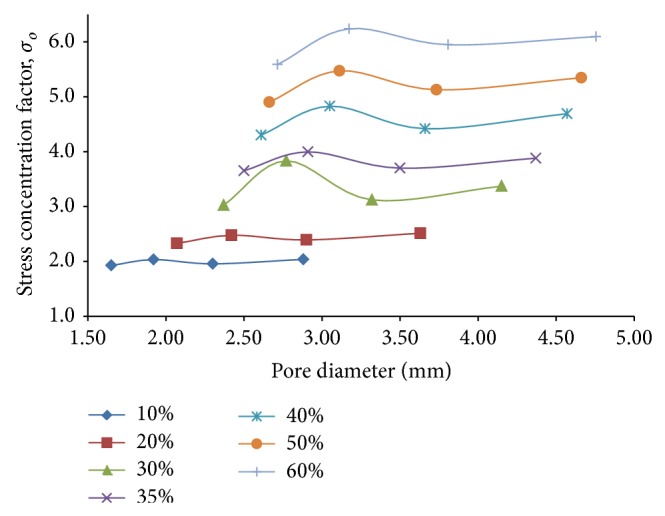
Plots depicting variation of stress concentration factor, *σ*_*o*_, against pore diameter for different percentages of porosity of 3D porous FE cantilever beam models subjected to combined loading action.

As far as the stress concentration effect around the pore periphery is concerned, it is found that the stress concentration factor, *σ*_*o*_, varies almost nominally with the pore diameter for a given porosity, for porosities in the lower ranges of 10% and 20%. But as the porosity percentage increases to higher levels, the variation of the stress concentration factor with pore diameter increases for a given porosity, the variation being around 12% of the mean stress concentration factor for porosities up to 60%. It can be clearly understood from Figure 15 that as the porosity is increased from 10% to 60%, the minimum stress concentration factor for a given porosity is found to increase from 2 to approximately 4.5. For a porosity of 60% that offers maximum overall stiffness reduction, it is seen that the lower values of stress concentration at around 4.3 are achieved for pore diameters in the two different regions of 2.6 mm and 3.6 mm. Thus, as far as stress concentration is concerned, both of these pore diameters may be recommended as equally favorable.

**Figure 16:**
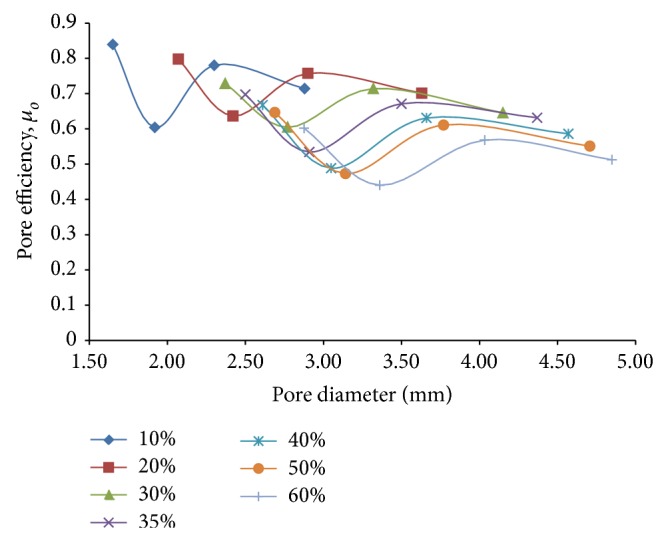
Plots depicting variation of pore efficiency under combined loading, *μ*_*o*_, against pore diameter for different percentages of porosity of 3D porous FE cantilever beam models subjected to combined loading action.

Finally, in order to optimize the effects of decreasing overall stiffness with increasing stress concentration against porosity, a new optimizing parameter similar to the one introduced for flexural loading response, called pore efficiency under combined loading, *μ*_*o*_ = 1/(*K*_*o*_ *σ*_*o*_) is introduced and examined in Figure 16. As the expression suggests, a higher value of pore efficiency indicates a greater suitability of a particular pore diameter over the other diameters. The plots of this parameter for different porosities (Figure 16) reveal that, for a given porosity, the pore efficiency varies appreciably with pore diameter, attaining their minimum values for pore diameters in the proximity of 3 mm for the higher porosities of 30% to 60%. But as the pore diameter is varied across different porosities, the values of maximum pore efficiency for a given porosity decrease steadily from the porosity of 10% to 60%. But since the decrement of the same with increasing porosity is not appreciably steep, it may be inferred that the conflicting effects of decreasing stiffness and increasing stress concentration compensate each other to a certain extent to keep the value of their product, as used in the optimizing parameter, from increasing by a substantial amount. As our primary objective lies in minimizing the stiffness of the implant material without letting the stress concentration exceed allowable limits, a check needs to be performed so as to determine whether maximum stiffness reduction for the higher porosities of 35% and 60% can be achieved while keeping the maximum von Mises stress values for combined loading within permissible limits.

Accordingly, it is observed that for a pore diameter in the proximity of 3.65 mm for 60% porosity, the stress concentration factor equals 4.5 approximately. Since the maximum von Mises stress that is generated in the region of interest (where porosity is going to be introduced) of the solid hip implant model as per [1] is around 93 N/mm^2^, a maximum von Mises stress of around 418.5 MPa is expected to arise around the pore periphery for the afore specified pore diameter and porosity. On taking the yield strength value of 895 MPa for annealed Ti-6Al-4V alloy under room temperature conditions, an approximate factor of safety of 2.14 is thus obtained for the suggested value of pore diameter. Since, during stumbling action, hip joint contact forces approximately twice as high as the forces under normal gait conditions might arise [19], this factor of safety is found to be on the conservative side and thus safe for design purposes.

**Figure 17:**
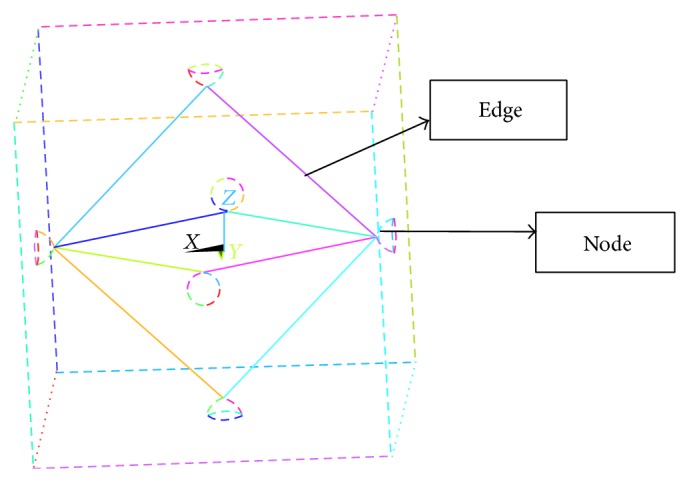
Octahedron model.

**Figure 18:**
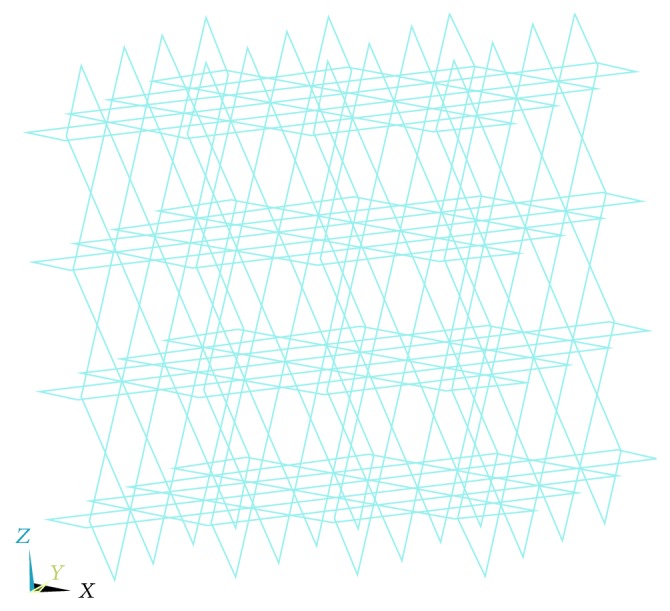
Octahedron model: displacement taken about the *z*-axis.

For the octahedrons model (Figures 17 and 18) we have gone through analysis with cubic and parallelepiped models. Here we also applied load with three types loading condition keeping all the parameters in mind.

## 7. Conclusion

The plots that have been obtained for the different kinds of loading (compressive, flexural, and combined) depicting the variation of the relative stiffness and stress concentration factor as well as the optimizing parameter and pore efficiency suggest that a pore diameter of 3.65 mm for 40% porosity produces the most favorable results as far as optimizing the stiffness and stress concentration parameters are concerned. Accordingly, a pore diameter of 3.65 mm giving rise to 40% porosity is recommended for a square array spherical pore distribution pattern in the region below the neck of the designed hip implant [1]. On incorporation of this optimum pore diameter of 3.65 mm in the porous region of the hip implant, a stiffness reduction of up to 65% that of the fully dense solid hip implant may be expected.

**Figure 19:**
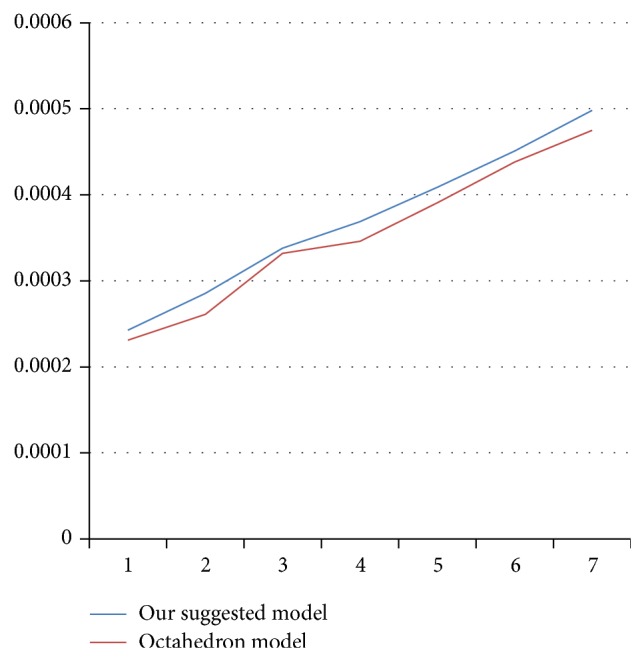
Showing that displacements are almost the same.

The displacement about the *z*-axis (Figure 19) is taken for the octahedron model of the top node. The result shows that displacement about *z*-axis for the both cases is almost the same. Therefore we would also suggest here that the octahedron model might be difficult to fabricate.

In the case of 60% of porosity the pore diameter of 4 mm would be the optimized diameter considering all the parameters. If the pore diameter was bigger, then there could be chances of crack development due to loading. Therefore the preferred porosity is 40%.

Although our present investigation was performed to address the biocompatibility concerning the design of hip implants, the plots that have been obtained in the process depicting the variation of the stiffness and stress quantities are all relative in nature. This means that their nature as well as the deductions that could be drawn from them hold true irrespective of the magnitude of loading that the implant is to be subjected to. This makes these plots equally usable for the design of pore diameters in the porous regions of implants other than hip implants that are subjected to similar loading and boundary conditions. Also, the plots that are obtained from the response of the porous material under the separate actions of flexural and compressive loading may be used instead of the plots obtained for combined loading response, where either of these loading cases is predominant over the other, unlike the case for hip and femoral implants. It is also noteworthy to mention here that the results and conclusions that are deduced from the present investigation alone do not ensure a perfect degree of biocompatibility of the implant. Proper consideration also needs to be given to the complications that might arise during fabrication of the porous material in the form of notches and imperfections that are capable of producing additional stress localization around the pores, as well as to the exact geometry of the implant which might entail additional considerations and design restrictions with respect to the pore distribution and morphology, before arriving at a specific diameter.

The future scope of work includes investigating the effects of notches and imperfections that might arise around the pore periphery of the porous implants during their fabrication procedure. Since the current undertaking deals with spherical porous inclusions only, a similar kind of investigation may also be performed with ellipsoidal pores, which might additionally help us to obtain an anisotropic mechanical response from the implant material, if and where it is necessary. Though, the feasibility of fabricating ellipsoidal pores using conventional pore manufacturing techniques, with respect to the degree of precision that can be attained during manufacturing, needs to be assessed first before conducting such an investigation. The present computational study may serve as a reference for these purposes.

## Figures and Tables

**Table 1:** Pore diameters (in mm) of 3D porous beam models used for different porosity percentages.

Porosity in %	Pore diameters in mm.			
10	2.88	2.3	1.92	1.65
20	3.63	2.9	2.42	2.07
30	4.15	3.32	2.77	2.37
35	4.37	3.5	2.91	2.5
40	4.57	3.66	3.05	2.61
50	4.66	3.8	3.17	2.71
60	4.7	3.95	3.29	2.82

## References

[1] Chatterjee S., Majumder S., Mondal P., Patwari M., Saha B., Roy Chowdhury A. Effective stiffness of customized hip implants incorporation of cavity.

[2] Niinomi M. (2008). Mechanical biocompatibilities of titanium alloys for biomedical applications. *Journal of the Mechanical Behavior of Biomedical Materials*.

[3] Ryan G. E., Pandit A. S., Apatsidis D. P. (2008). Porous titanium scaffolds fabricated using a rapid prototyping and powder metallurgy technique. *Biomaterials*.

[4] Khan S. N., Tomin E., Lane J. M. (2000). Clinical applications of bone graft substitutes. *Orthopedic Clinics of North America*.

[5] Long M., Rack H. J. (1998). Titanium alloys in total joint replacement—a materials science perspective. *Biomaterials*.

[6] Spoerke E. D., Murray N. G., Li H., Brinson L. C., Dunand D. C., Stupp S. I. (2005). A bioactive titanium foam scaffold for bone repair. *Acta Biomaterialia*.

[7] Banhart J. (2001). Manufacture, characterisation and application of cellular metals and metal foams. *Progress in Materials Science*.

[8] Parthasarathy J., Starly B., Raman S., Christensen A. (2010). Mechanical evaluation of porous titanium (Ti6Al4V) structures with electron beam melting. *Journal of the Mechanical Behavior of Biomedical Materials*.

[9] Bram M., Schiefer H., Bogdanski D., Köller M., Buchkremer H. P., Stöver D. Evaluation of mechanical and biological properties of highly porous titanium parts.

[10] Shen H., Oppenheimer S. M., Dunand D. C., Brinson L. C. (2006). Numerical modeling of pore size and distribution in foamed titanium. *Mechanics of Materials*.

[11] Liu P. S. (2007). A new analytical model about the relationship between nominal failure stresses and porosity for foamed metals under biaxial tension. *Materials and Design*.

[12] Liu P. S. (2010). Mechanical relations for porous metal foams under several typical loads of shearing, torsion and bending. *Materials Science and Engineering A*.

[13] Liu P. S. (2011). Failure by buckling mode of the pore-strut for isotropic three-dimensional reticulated porous metal foams under different compressive loads. *Materials and Design*.

[14] Roberts A. P., Garboczi E. J. (2002). Elastic properties of model random three-dimensional open-cell solids. *Journal of the Mechanics and Physics of Solids*.

[15] Bastawros A.-F., Bart-Smith H., Evans A. G. (2000). Experimental analysis of deformation mechanisms in a closed-cell aluminum alloy foam. *Journal of the Mechanics and Physics of Solids*.

[16] Ford C. M., Gibson L. J. (1998). Uniaxial strength asymmetry in cellular materials: an analytical model. *International Journal of Mechanical Sciences*.

[17] Duda G. N. (1996). *Influence of muscle forces on the internal loads in the femur during gait [Ph.D. thesis]*.

[18] Niu W., Gill S., Dong H., Bai C. (2010). A two-scale model for predicting elastic properties of porous titanium formed with space-holders. *Computational Materials Science*.

[19] Bergmann G., Graichen F., Rohlmann A. (2004). Hip joint contact forces during stumbling. *Langenbeck's Archives of Surgery*.

